# 

**Published:** 1870-10

**Authors:** 


### BOOK NOTICES.

"Home Life."—We have for a long time been carefully laying aside the successive numbers of *The Boston Journal of Chemistry*, devoted to the science of Home Life, Arts, Agriculture and Medicine, published monthly at $1.50 a year; and since commencing this article, we have noticed for the first time that it is published by our own publishers, Hurd & Houghton, 13 Astor Place, New York. We have for some months been waiting for time to look over the items leisurely, so many of them merit preservation. Despite its scientific title, like our old favorite, *The Scientific American*, it is a most valuable family paper, and will in various ways save more than its price by the value of its practical household and family hints.

"The Garden of Sorrow, or the Ministry of Tears," by the Rev. John Atkinson, M.A; Carlton & Lanahan, publishers in New York; E. Thomas, San Francisco; Hitchcock & Walden, Cincinnati. This is a book got up in elegant style; its matter commends itself to all of woman born—to all who pass through trouble, and sorrow, and tears; its chapters speak beautifully of Gethsemane, and of Him who there sorrowed unto death; of a sorrowing Church; of sorrowing daughters; of springs of sorrow; of despondency; of temptation; of chastening; of poverty and misfortune; of the sick-room; the chamber of death; death of children; graves of our dead; loneliness; compensation; consummation.

The American Institute, on Third Avenue and 63d Street, New York, is now in full and successful operation, and thousands of intelligent persons are visiting it every day, showing to society, to the country, and to the world, as no other exhibition can show, what a power for good, for elevation, is the mechanics, the artisans, and the inventors of our nations. The New York *Tribune* justly says:

"The grand exhibition at the Empire Rink is rapidly improving in every respect. In the machinery department, a full line of shafting is already in complete working order, and numerous machines will soon be in operation. Among the more noteworthy inventions, Daniel Jackson, of Broadway, exhibits Strong's combined letter and milk box; and the Knox fluting machine, with new and ingenious diamond and concave roller, and a spring lever which holds up the upper roller, and gives the operator the use of both hands. Twelve thousand of these machines have been sold within the year, and they are in use at Lord & Taylor's, the Astor House, and by many other leading firms and hotels. The Cole fluting machine, exhibited by Mrs. Cole herself, has rollers regulated by a self-acting lever-power, and conforming to all kinds of work without the aid of hand or foot. Mrs. Knox and Mrs. Cole are the pioneers in American fluting, and their machines are both worth careful inspection. I. & J. A. Josephs, of Broome-street, exhibit Simmon's syphon and reversible filterers. The latter consists simply of a coiled spring, wound with flannel, and contained in a small cylindrical case. One of these, fitted to a hydrant, at an expense of $2 or $3, will filter every drop of water required by a family, removing mechanically all impurities, and rendering it perfectly clear and fit for any use. It is very simple in construction, and the impurities collected are readily removed by merely reversing it once a day. In the syphon filterer a quantity of sand, placed between two layers of wool, is the filtering material, and the water, oil, or other fluid, passes up instead of down. T. McElroy, of Perry-st., exhibits an oculist's operating chair, with ingenious appliances for supporting and holding the head, raising and lowering the body, and adjusting the seat to a patient of any height; a surgical operating chair, with important arrangements for facilitating lithotomy and speculum examination; an invalid elevating bedstead, for raising the patient while the bed is made, or for elevating only the head, the knees, or the feet; and an invalid fracture bedstead, with movable rests for the four limbs, operating singly or altogether, and an extension appliance, for extending the muscles and preventing rotation in case of fractures. The chairs are new inventions, and the bedsteads have been much improved.

John Matthews, of First-ave., exhibits a complete array of soda-water apparatus, including machines for making, dispensing, and bottling. His two soda founts are among the finest features of the Fair. The "Snow Queen," in the centre of the Rink, is probably the most perfect, beautiful, and costly soda fountain ever erected in the United States, or perhaps in the world. It is 15 feet in diameter, with eight sides, each six feet long.

New Article of Food.—For twenty-five cents you can buy of your druggist or grocer a package of sea moss farine, manufactured from pure Irish moss or carrageen, which will made sixteen quarts of blanch mange and a like quantity of puddings, custards, creams, charlotte russe, and is among the cheapest and most healthful articles for the table.

### INDEX OF HALL'S HEALTH TRACTS.

Containing 350 Health Tracts on the following subjects, Price $2.00.

Aphorisms Physiolog'l.
Apples.
Antidote to Poisons,
Acre, One.
Apoplexy.
Burying Alive.
Baths and Bathing.
Beards,
Bites and Burns.
Backbone,
Beauty a Medicine.
Best Day.
Baldness.
Burning to Death.
Bilious Diarrhea.
Balm of Gilead.
Bread.
Cold Cured.
" Neglected.
" Avoided.
" Nothing but a.
" In the Head.
" How Taken.
" Catching.
Consumption.
Coffee-Drinking.
Catarrh.
Checking Perspiration.
Centenarian.
Child-Bearing
Children's Eating.
" Feet.
Children Corrected.
" Dirty.
Coal-Fires.
Cute Things.
Coffee Poisons.
Clothing, Flannel.
" Woolen.
" Changing.
Cholera.
Clergymen.
Cancer.
Corn-Bread.
Convenient Knowledge.
Charms.
Cooking Meats.
Cheap Bread.
Church Ventilation.
Cough.
Dyspepsia
Drinking.
Diet for the Sick.
Deafness.
Debt, a Death!
Diarrhea.
Death.
Dying Easily.
Drowning.
Diptheria.
Dysentery.
Disinfectants.
Death Rate.
Deranged.
Digestibility of Food.
Dirty Children.
Drugs and Druggery.
Eyes, Care of.
" Weak.
" Failing.
Erect Position.
Eating.
" Wisely.
Eat, How to.
" What and when to.
Eating Habits.
" Great.
" Curiosities of.
" Economical.
Emanations.
Elements of Food.
Fruits, Uses of.
Flannel Wearing.
Follies, Fifteen.
Fire-Places.
Fifth Avenue Sights.
Food and Health.
Feet.
Fetid Feet.
Food, Nutritiousness of.
" its Elements.
Greed of Gold.
Genius, Vices of.
Great Eaters.
Gruels and Soups.
Hair Wash.
Health a Duty.
" Observances.
" Essentials.
" Theories.
Hydrophobia.
Headache.
Housekeeping.
Housewifery.
Household Knowledge.
Habit.
Home, Leaving.
Happiest, Who are.
Hunger.
Inconsiderations.
Ice, Uses of.
Inverted Toe-Nail.
Insanity.
In the Mind.
Kindness Rewarded.
Law of Love.
Longevity.
Life Wasted.
Loose Bowels.
Leaving Home.
Logic Run Mad.
Medicine Taking.
Memories.
Music Healthful.
Milk, its Uses.
Miasm.
Marriage.
Morning Prayer.
Month Malign.
Mental Ailments.
Mind Lost.
Medical Science.
Neuralgia.
Nursing Hints.
Nervous Debilities.
Old Age Beautiful.
One Acre.
One by One.
Obscure Diseases.
Precautions.
Presence of Mind.
Premonitions.
Private Things.
Poisons and Antidotes
Pain.
Peaceless.
Preserves.
Parental Trainings.
Philosophy.
Physiological Items.
Posture in Worship.
Physician, Faithless.
Popular Fallacies.
Punctuality.
Providence.
Rheumatism.
Read and Heed.
Resignation.
Restless Nights.
Recreation, Summer
Restlessness
Sitting Erectly.
Shoes Fitting.
Sabbath.
Sour Stomach.
Sick Headache.
Sunshine
Skating.
Suppers, Hearty.
Soldiers Remembered.
" Cared for
" Health.
" Items.
" All.
Serenity.
Sores.
Sunday Dinners.
Small-Pox.
Sleep and Death.
Spot the One.
Specifics.
Spring-Time.
Summer Drinks.
Sickness not Causeless
Sayre, the Banker.
September Malign.
Summer Mortality
Stammering.
Soups and Gruels.
Sick School-Girl.
Stomach's Appeal.
Sleep,
Summerings.
Study, Where to.
Salt Rheum.
Sordid.
Traveling Hints.
Three Ps.
Teeth.
Toe-Nail, Inverted.
Thankful Ever.
Urination.
Valuable Knowledge
Vaccination.
Ventilation.
Vermin Riddance.
Winter Rules.
Walking.
Warning Youth.
Woman's Beauty
Whitlow.
Whitewashes.
Worth Remembering.
Worship, Public.
" Posture in
" Without Price
Weather Signs.
Weather and Wealth.
Warmth and Strength.
Worth Knowing.

